# Special Issue “SARS-CoV-2: Epidemiology and Pathogenesis”: Editorial

**DOI:** 10.3390/microorganisms11040927

**Published:** 2023-04-03

**Authors:** Paolo Calistri, Harsharn Gill, Alessio Lorusso

**Affiliations:** 1 Istituto Zooprofilattico Sperimentale dell’Abruzzo e del Molise “G. Caporale”, 64100 Teramo, Italy; 2 School of Science, STEM College, RMIT University, Melbourne, VIC 3000, Australia

Since its emergence in 2019 in Wuhan City, Hubei Province, China, SARS-CoV-2 has spread across hundreds of countries and all continents. As the virus is easily transmitted from person to person, it has caused the most serious public health emergency that the world has faced since the Spanish flu.

Over the past three years of the COVID-19 pandemic, a large number of scientific papers have been published in various journals in an effort to quickly fill the fundamental knowledge gap on this new virus. This Special Issue was a small attempt to provide a contribution to the scientific debate on SARS-CoV-2 virus infection and allow scientists and professionals actively working on the surveillance, prevention, or control of the COVID-19 disease to share their experiences and findings with the rest of the scientific community.

This Special Issue, comprising 22 articles, aimed to highlight the most recent outcomes of scientific research on the pathogenesis, epidemiology, and diagnosis of SARS-CoV-2 infection, including aspects related to animal reservoirs, virulence factors, viral evolution, control measures applicable at local and international levels, and the impact on global and local economies.

The contributions came from all continents (15 from Europe, 3 from America, 2 from Asia, 1 from Africa, and 1 from Australia), with authors from 14 different countries; thus, these articles represent a great variety of situations and experiences. Concerning the topics, the majority of the papers (*n* = 11) dealt with the epidemiology of SARS-CoV-2 infection in various contexts, while 10 articles focused on specific aspects characterizing the pathogenesis of the disease. One paper provided interesting insights into vaccines.

Looking at the affiliations of the authors, a wide spectrum of disciplines and institutions are represented, including medical institutes, universities, hospitals, genetic and research centers, public health organizations, and veterinary institutions. This multidisciplinary composition of the contributors to this Special Issue provides the readers with multiple and original views and approaches, enriching the scientific debate on SARS-CoV-2 epidemiology and pathogenesis.

It is noteworthy that most papers in this Special Issue, regardless if the topics address epidemiology or pathogenesis, are aimed at inferring useful information for improving the surveillance, diagnosis, prevention, and control of COVID-19, and help to elucidate the main transmission routes and pathogenetic mechanisms.

Concerning papers dealing with pathogenetic mechanisms, Kalkeri et al. [1] developed a BSL-2 pseudovirus-based neutralization assay (PBNA) useful for measuring the neutralization ability of candidate vaccines in both preclinical models and clinical trials. Another paper attempted to elucidate the structural, surface, and functional properties of viral proteins [2]. Other articles investigated the persistence of SARS-CoV-2 viral RNA in dead patients [3]; the efficacy of antiviral agents against SARS-CoV-2 [4]; the prognostic value of eosinopenia [5]; the interactions between human and viral proteins [6]; the cross-reaction of SARS-CoV-2 with antibodies against other human coronaviruses and its significance for the establishment of the immunity [7]; the whole genome intra-host variability of SARS-CoV-2 in the upper and lower respiratory tract in patients [8]; the immune response, inflammatory reactions, and viral replication in COVID-19 disease [9]; and the pathogenicity and transmissibility of the Delta and Lambda variants [10].

Some papers dealing with epidemiological features focused on the temporal and/or geographic distribution of SARS-CoV-2 lineages [11,12,13,14], whereas others explored the epidemiological significance of diagnostic findings [15,16,17,18]. Two papers presented different approaches for predicting the occurrence of infection [19,20], whereas one article discussed the main biological, ecological, and economic drivers facilitating the emergence of SARS-CoV-2 and its spread worldwide [21]. Finally, one paper explored the various possible candidate vaccines and their possible use on a large scale [22].

It is to be noted that the concerted research efforts by the scientific community globally, supported by substantial investments by governments and other organizations across the globe, have been responsible for a broad range of innovations related to virus characterization, testing, and sequencing, as well as disease pathogenesis and the development of effective vaccines and a few drugs in a record time. Lessons learned from this pandemic puts us in good stead to respond rapidly and effectively to new viruses and/or pandemics in the future.

## References

[1] Kalkeri R., Cai Z., Lin S., Farmer J., Kuzmichev Y.V., Koide F. (2021). SARS-CoV-2 Spike Pseudoviruses: A Useful Tool to Study Virus Entry and Address Emerging Neutralization Escape Phenotypes. Microorganisms.

[2] Areo O., Joshi P.U., Obrenovich M., Tayahi M., Heldt C.L. (2021). Single-Particle Characterization of SARS-CoV-2 Isoelectric Point and Comparison to Variants of Interest. Microorganisms.

[3] Servadei F., Mauriello S., Scimeca M., Caggiano B., Ciotti M., Anemona L., Montanaro M., Giacobbi E., Treglia M., Bernardini S. (2021). Persistence of SARS-CoV-2 Viral RNA in Nasopharyngeal Swabs after Death: An Observational Study. Microorganisms.

[4] Jo S., Kim S., Yoo J., Kim M.-S., Shin D.H. (2021). A Study of 3CLpros as Promising Targets against SARS-CoV and SARS-CoV-2. Microorganisms.

[5] Le Borgne P., Abensur Vuillaume L., Alamé K., Lefebvre F., Chabrier S., Bérard L., Haessler P., Gennai S., Bilbault P., Lavoignet C.-E. (2021). Do Blood Eosinophils Predict in-Hospital Mortality or Severity of Disease in SARS-CoV-2 Infection? A Retrospective Multicenter Study. Microorganisms.

[6] Cardon T., Fournier I., Salzet M. (2020). SARS-Cov-2 Interactome with Human Ghost Proteome: A Neglected World Encompassing a Wealth of Biological Data. Microorganisms.

[7] Simula E., Manca M., Jasemi S., Uzzau S., Rubino S., Manchia P., Bitti A., Palermo M., Sechi L. (2020). HCoV-NL63 and SARS-CoV-2 Share Recognized Epitopes by the Humoral Response in Sera of People Collected Pre- and during CoV-2 Pandemic. Microorganisms.

[8] Rueca M., Bartolini B., Gruber C., Piralla A., Baldanti F., Giombini E., Messina F., Marchioni L., Ippolito G., Di Caro A. (2020). Compartmentalized Replication of SARS-Cov-2 in Upper vs. Lower Respiratory Tract Assessed by Whole Genome Quasispecies Analysis. Microorganisms.

[9] Zafer M., El-Mahallawy H., Ashour H. (2021). Severe COVID-19 and Sepsis: Immune Pathogenesis and Laboratory Markers. Microorganisms.

[10] Moghaddar M., Radman R., Macreadie I. (2021). Severity, Pathogenicity and Transmissibility of Delta and Lambda Variants of SARS-CoV-2, Toxicity of Spike Protein and Possibilities for Future Prevention of COVID-19. Microorganisms.

[11] Klempt P., Brzoň O., Kašný M., Kvapilová K., Hubáček P., Briksi A., Bezdíček M., Koudeláková V., Lengerová M., Hajdúch M. (2021). Distribution of SARS-CoV-2 Lineages in the Czech Republic, Analysis of Data from the First Year of the Pandemic. Microorganisms.

[12] Widera M., Mühlemann B., Corman V., Toptan T., Beheim-Schwarzbach J., Kohmer N., Schneider J., Berger A., Veith T., Pallas C. (2021). Surveillance of SARS-CoV-2 in Frankfurt am Main from October to December 2020 Reveals High Viral Diversity Including Spike Mutation N501Y in B.1.1.70 and B.1.1.7. Microorganisms.

[13] Goncalves Cabecinhas A., Roloff T., Stange M., Bertelli C., Huber M., Ramette A., Chen C., Nadeau S., Gerth Y., Yerly S. (2021). SARS-CoV-2 N501Y Introductions and Transmissions in Switzerland from Beginning of October 2020 to February 2021—Implementation of Swiss-Wide Diagnostic Screening and Whole Genome Sequencing. Microorganisms.

[14] Viedma E., Dahdouh E., González-Alba J., González-Bodi S., Martínez-García L., Lázaro-Perona F., Recio R., Rodríguez-Tejedor M., Folgueira M., Cantón R. (2021). Genomic Epidemiology of SARS-CoV-2 in Madrid, Spain, during the First Wave of the Pandemic: Fast Spread and Early Dominance by D614G Variants. Microorganisms.

[15] Calistri P., Danzetta M., Amato L., Cito F., Di Giuseppe A., Zenobio V., Morelli D., Puglia I., Caporale M., Scialabba S. (2021). Epidemiological Significance of SARS-CoV-2 RNA Dynamic in Naso-Pharyngeal Swabs. Microorganisms.

[16] Muñoz-Medina J., Grajales-Muñiz C., Salas-Lais A., Fernandes-Matano L., López-Macías C., Monroy-Muñoz I., Santos Coy-Arechavaleta A., Palomec-Nava I., Duque-Molina C., Madera-Sandoval R. (2021). SARS-CoV-2 IgG Antibodies Seroprevalence and Sera Neutralizing Activity in MEXICO: A National Cross-Sectional Study during 2020. Microorganisms.

[17] D’Ardes D., Pontolillo M., Esposito L., Masciarelli M., Boccatonda A., Rossi I., Bucci M., Guagnano M., Ucciferri C., Santilli F. (2020). Duration of COVID-19: Data from an Italian Cohort and Potential Role for Steroids. Microorganisms.

[18] Danzetta M., Amato L., Cito F., Di Giuseppe A., Morelli D., Savini G., Mercante M., Lorusso A., Portanti O., Puglia I. (2020). SARS-CoV-2 RNA Persistence in Naso-Pharyngeal Swabs. Microorganisms.

[19] Savini L., Candeloro L., Calistri P., Conte A. (2020). A Municipality-Based Approach Using Commuting Census Data to Characterize the Vulnerability to Influenza-Like Epidemic: The COVID-19 Application in Italy. Microorganisms.

[20] Ilie O., Cojocariu R., Ciobica A., Timofte S., Mavroudis I., Doroftei B. (2020). Forecasting the Spreading of COVID-19 across Nine Countries from Europe, Asia, and the American Continents Using the ARIMA Models. Microorganisms.

[21] Calistri P., Decaro N., Lorusso A. (2021). SARS-CoV-2 Pandemic: Not the First, Not the Last. Microorganisms.

[22] Loo K., Letchumanan V., Ser H., Teoh S., Law J., Tan L., Ab Mutalib N., Chan K., Lee L. (2021). COVID-19: Insights into Potential Vaccines. Microorganisms.

